# Lower urinary tract and bowel dysfunction in spinocerebellar ataxias

**DOI:** 10.1002/acn3.51266

**Published:** 2020-12-18

**Authors:** Joana Afonso Ribeiro, Sara Simeoni, Lorenzo De Min, Tomoyuki Uchiyama, Yu Tung Lo, Nita Solanky, Hector Garcia‐Moreno, Paola Giunti, Jalesh N. Panicker

**Affiliations:** ^1^ Ataxia Service Department of Clinical and Movement Neurosciences and Department of Neurogenetics The National Hospital for Neurology and Neurosurgery and UCL Queen Square Institute of Neurology Faculty of Brain Sciences University College London London United Kingdom; ^2^ Neurology Department Child Development Centre Coimbra’s Hospital and University Centre Coimbra Portugal; ^3^ Department of Uro‐Neurology The National Hospital for Neurology and Neurosurgery, and UCL Queen Square Institute of Neurology Faculty of Brain Sciences University College London London United Kingdom; ^4^ Envida Maastricht The Netherlands; ^5^ Department of Neurology School of Medicine International University of Health and Welfare/International University of Health and Welfare Ichikawa and Narita Hospital Chiba Japan; ^6^ Department of Neurosurgery National Neuroscience Institute Singapore Singapore

## Abstract

**Background:**

Little information is available in spinocerebellar ataxias (SCAs) regarding pelvic organ symptoms. The aim of this study was to characterize the lower urinary tract (LUT) and bowel dysfunction in autosomal dominant spinocerebellar ataxias.

**Methods:**

Patients with confirmed SCAs attending a tertiary care service were approached about LUT and bowel complaints, and completed validated questionnaires: urinary symptom profile (USP), Qualiveen‐Short form, International Prostate Symptom Score, and Neurogenic Bowel Dysfunction Score. SCA3 and SCA7 patients with urological complaints additionally underwent urodynamic studies (UDS). Patients’ characterization included demographic, clinical (Scale for the Assessment and Rating of Ataxia (SARA), Inventory of Non‐Ataxia Signs (INAS)), and genetic variables. Descriptive and comparative analyses were performed.

**Results:**

Fifty‐one patients participated: SCA1 (n = 4), SCA2 (n = 11), SCA3 (n = 13), SCA6 (n = 17), and SCA7 (n = 6). The prevalence of self‐reported LUT symptoms was 60.8% (n = 31), whereas LUT symptoms was reported in 86.3%(n = 44) using the USP. Both storage and voiding symptoms were reported, urinary frequency and urgency being the most frequent (n = 34, 68%). Although LUT symptoms were most often classed as mild (n = 27, 61.4%), they impacted QoL in 38 patients (77.6%). Of these, 21 (55.3%) were not on pharmacological treatment for urinary dysfunction. Most common abnormalities in UDS (n = 14) were detrusor overactivity (storage phase) and detrusor underactivity (voiding phase). Bowel symptoms were less common (31.4%, n = 16) and of mild severity.

**Conclusion:**

LUT symptoms are prevalent in SCA patients and impact QoL, whereas bowel symptoms tend to be mild. These symptoms are overlooked by patients and physicians due to the complexity of neurological involvement in SCA, and therefore a multidisciplinary management approach should be adopted.

## Introduction

Autosomal dominantly inherited spinocerebellar ataxias (SCAs) are a genetically heterogeneous group of neurodegenerative disorders clinically characterized by a progressive deterioration in gait and balance and variably associated with extrapyramidal, bulbar, spinal cord, and peripheral nervous system involvement.^1^, ^2^ These are most commonly due to triplet repeat expansion in coding regions, and, in the case of CAG repeats expansion, differences in repeat size are contributors to the variability in phenotypical presentation, disease severity, and progression.^3^, ^4^, ^5^ Worldwide, SCA3 is most common and probably accounts for more than half of the affected families, followed by SCA 2, 6, 1, and 7.^2^, ^3^, ^4^ Phenotypically, SCA6 is considered a predominantly cerebellar ataxia, whereas SCA1, 2, 3, and 7 present additional nonataxia symptoms.^5^, ^6^ Also, patients with SCA6 tend to present usually after 50 years of age, whereas the disease usually begins between the third and the forth decade of life in the other SCAs.^6^

Lower urinary tract (LUT) dysfunction has been reported in a large European cohort study of patients with SCA1, SCA2, SCA3, and SCA6, ranging between 31 and 46%.^7^ Other larger studies in SCA patients and smaller studies exploring nonmotor symptoms in SCA2, SCA3, and SCA6 patients have confirmed LUT dysfunction,^8^, ^9^, ^10^, ^11^, ^12^, ^13^, ^14^, ^15^ however, little is known about the relationship between LUT and neurological dysfunction and information is limited to SCA3 about bowel dysfunction.^11^, ^12^ The aim of this study was to evaluate the prevalence of LUT and bowel symptoms in the common autosomal dominant inherited spinocerebellar ataxias, assess impact on quality of life, and explore the association between LUT symptoms, neurological features, and genetic mutations.

## Methods

Patients (>18 years of age) with genetically confirmed spinocerebellar ataxias with (CAG) trinucleotide repeat—types 1, 2, 3, 6, and 7 ‐ attending a national Ataxia Centre at a tertiary academic hospital were evaluated. All patients had a genetically confirmed diagnosis of SCA by genetic analysis of the heterozygous mutations responsible for each SCA subtype ‐*ATXN1* for SCA1, *ATXN2* for SCA2, *ATXN3* for SCA3, *CACNA1A* for SCA6, and *ATXN7* for SCA7. The (CAG)_n_ expansion size for the normal and expanded allele was determined by polymerase chain reaction by an accredited genetic laboratory. Patients were approached either during their clinical appointment at the Ataxia Centre or through telephone, and those interested in participating underwent a clinical assessment enquiring about duration of neurological symptoms, LUT and bowel complaints, treatments received, and completed the questionnaires. The interval between questionnaire completion and clinical evaluation was less than 6 months. The survey was registered with the local Divisional Quality Safety and Governance Board as a service evaluation.

The following scales and questionnaires were administered:

‐ Scale for the Assessment and Rating of Ataxia (SARA) is a widely accepted and validated score to measure ataxia severity, consisting of a composite score of cerebellar manifestations of ataxia, ranging from 0 to 40, with higher scores reflecting greater severity of ataxia.^16^, ^17^
‐ Inventory of Non‐Ataxia Symptoms (INAS)^18^ is an inventory of symptoms ‐ motor, sensory, ocular signs, reported abnormalities, and cognitive impairment. Question 28 enquires about self‐reported urinary dysfunction which is graded as mild, moderate, or severe/requiring catheterization. INAS scores were further divided into pyramidal features (hyperreflexia, extensor plantar responses, and spasticity), extrapyramidal (rigidity, resting tremor, dystonia, and chorea) and peripheral nervous system involvement (distal hypo/arreflexia, distally impaired vibration sense, atrophy, or fasciculations). The highest count on the INAS is 15, because the question regarding urinary dysfunction was excluded from the analysis.
‐ Urinary Symptom Profile (USP)^19^ is a validated questionnaire of LUT symptoms that includes sub‐sections covering stress urinary incontinence (SUI), overactive bladder (OAB), and low stream (LS) symptoms. Maximum scores, suggesting worse symptoms, are 21, 9, and 9, respectively.
‐ International Prostate Symptom Score (I‐PSS) is a standardized validated questionnaire of LUT symptoms that measures the severity of urinary symptoms in both men and women.^20^ Scores can range between 0 and 35, and severity of LUT symptoms can be defined as mild (score <8), moderate (score 8‐19), or severe (score >20).
‐ SF‐Qualiveen is a health‐related quality of life (QoL) questionnaire for urinary symptoms,^21^ and has been validated for neurological disorders. The questionnaire is composed of 8 items distributed between four domains: “bother with limitations,” “frequency with limitations,” “fears,” and “feelings”. Scores range between 0 and 4 and greater scores indicate a higher impact on QoL.
‐ Neurogenic Bowel Dysfunction (NBD) questionnaire is a validated questionnaire evaluating bowel symptoms in neurological patients.^22^ The scores are weighted according to impact on QoL, with a maximum score of 47, and can be graded according to severity: very minor (score 1‐6), minor (score 7‐9), moderate (score 10‐13), and severe (score >13).

Following existing clinical pathways,^23^ patients with significant urogenital or bowel complaints were referred to the Department of Uro‐Neurology and received a standard clinical assessment. They underwent urodynamics testing: noninvasive uroflowmetry, measurement of postvoid residual (PVR) volumes, and, when indicated, invasive multichannel cystometrography, which comprised filling cystometry (medium fill at 50 mL/min) and pressure‐flow studies (Medical Measurement Systems, Dover, NH, USA), which were performed according to International Continence Society Good Urodynamic Practices.^24^

### Statistical analysis

IBM SPSS Statistics (version 24) was used to perform the statistical analysis and descriptive analysis was conducted to calculate mean, median, interquartile range (IQR), and standard deviation (SD) of the patient demographic and clinical characteristics, and the scores from the questionnaires evaluating LUT and bowel symptoms. Qualitative variables, such as the presence or absence of symptoms were presented as percentages. Statistical significance for intergroup differences (including gender, different SCAs, and more detailed subgroups) was assessed with Pearson’s χ^2^ or Fisher exact test for categorical variables and with Student’s t or Mann‐Whitney’s U test for continuous variables. Correlations were investigated using Spearman rank correlation. To assess clinical determinants of LUT symptoms, a linear regression analysis was performed and age, gender, duration of ataxia, SARA score, and INAS count were included as covariates. Statistical significance was defined as *P* < 0.05.

## Results

### Demographics and clinical characterization

**Figure 1:**
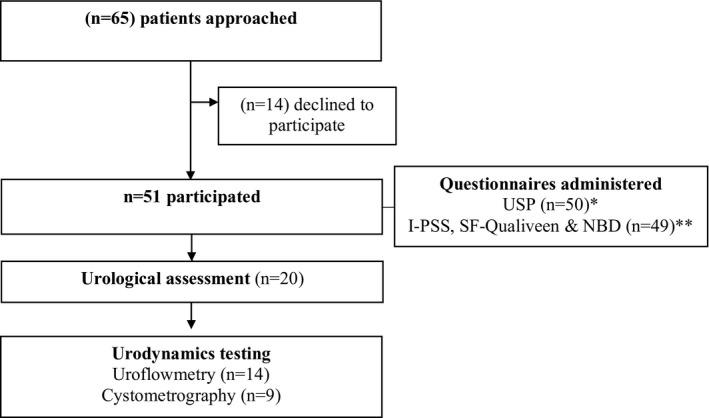
Overview of assessments. * indwelling catheter (n = 1) ** Incomplete questionnaire (n = 1). USP: Urinary symptom profile; I‐PSS: International Prostate Symptom Score; NBD: Neurogenic bowel dysfunction.

Fifty‐one patients were evaluated (Figure 1). Their demographic and clinical characteristics, according to SCA subtype, are shown in Table 1.

**Table 1:** Demographic and clinical characteristics of 51 patients with SCA.

	SCA1	SCA2	SCA3	SCA6	SCA7	TOTAL
n	4	11	13	17	6	51
Female gender (n,%)	1 (25.0)	4 (36.4)	9 (69.2)	11 (64.7)	3 (50.0)	28 (54.9)
Median age (IQR)	53.0 (32.5‐57.0)	45.0 (42‐62)	57.0 (37.0‐62‐0)	74.0 (65.5‐77.5)	51.0 (45.75‐63.50)	59.0 (45.0‐69.0)
Median age at ataxia onset (IQR)	47.0 (30.0‐47.0)	40.0 (31.0‐48.0)	40.0 (25.0‐52.0)	60.0 (52.5‐64.0)	39.5 (32.50‐44.75)	48.0 (38.75‐58.0)
Median duration of ataxia (IQR)	10.0 (7.75‐16.75)	7.0 (3.0‐17.0)	10.0 (5.0‐15.0)	15.0 (5.0‐15.0)	15.0 (8.75‐17.00)	10.0 (6.75‐17.25)
Mean (CAG)_n_ expanded*/normal	45.3/ 28.3	41.2/ 22.2	71.2/ 20.9	22.1/ 12.4	46.2/ 11.5	NA
Mean SARA score (SD)	10.9 (12.37)	11.4 (6.0)	17.0 (9.27)	12.0 (7.48)	15.9 (4.83)	13.5 (8.01)
Mean INAS count (SD)	5.0 (3.65)	3.1 (1.36)	5.5 (2.13)	2.2 (1.86)	2.7 (1.53)	3.6 (2.30)
Clinical evaluation (n,%)						
Pyramidal signs	4 (100.0)	3 (27.3)	8 (61.5)	5 (29.4)	4 (66.7)	24 (47.1)
Extrapyramidal	2 (50.0)	5 (45.5)	6 (46.2)	1 (5.9)	0 (0.0)	14 (27.5)
PNS	2 (50.0)	11 (100.0)	11 (84.6)	11 (64.7)	1 (16.7)	36 (70.6)

(CAG)_n_, trinucleotide expansion size; INAS, inventory of non‐ataxia signs; IQR, inter‐quartile range; PNS, peripheral nervous system. NA, not applicable; SARA, Scale for the assessment and rating of ataxia; SD, standard deviation. Age and duration of ataxia is depicted in years.

* Not available in three patients.

Median duration of ataxia was 10.0 years. All patients were heterozygous for a *CAG* repeat expansion for all the SCA included – mean *CAG* repeat length of each group is outlined in Table 1 (not available in 3 patients). The more common extracerebellar sign was reduced distal vibration sense (n = 32; 64%). The most common SCA subtype was SCA6, and a comparison between the clinical characteristics of the SCA6 patients and the other SCAs suggested that they were older (*P* < 0.001) and having less nonataxia features (*P* = 0.01). SCA1, SCA2, SCA3, and SCA7 patients had a similar age at ataxia onset. Comparison of demographic and clinical parameters between females and males showed no statistically significant differences.

### LUT Symptoms

60.8% (n = 31) of patients self‐reported urinary complaints during the clinical assessment (Table 2), with a median duration of symptoms of 8.0 years (range 1 to 51, IQR 3.0‐14.75). Patients with self‐reported LUT symptoms were significantly older (median age 62.0 vs. 54.0, *P* = 0.034) and had a more severe degree of ataxia (mean SARA score 16.13 vs. 9.53, *P* = 0.03), but there were no statistically significant differences regarding the presence of pyramidal, extrapyramidal, or neuropathic symptoms.

**Table 2:** Self‐reported bladder complaints and characterization of LUT symptoms according to the Urinary symptom profile questionnaire.

	SCA1	SCA2	SCA3	SCA6	SCA7	TOTAL
Self‐reported bladder complaints n (%)	2 (50.0)	6 (54.5)	8 (61.5)	10 (58.8)	5 (83.3)	31 (60.8)
Duration of bladder complaints Median (IQR)	2.0 (1.0‐2.0)	10.0 (2.5‐18.5)	3.5 (1.5‐10.8)	11.0 (5.5‐18.5)	13.0 (5.5‐19.0)	8.0 (3.0‐14.75)
USP (n = 50)* median (IQR)	9.50 (4.5‐10.75)	3.0 (0‐5.0)	10.0 (2.0‐13.0)	5.0 (1.5‐10.0)	6.5 (2.25‐11.0)	5.0 (2.0‐10.25)
SUI	0 (0‐1.5)	0 (0‐0)	0.5 (0‐2.75)	0 (0‐1.5)	0.5 (0‐2.0)	0.0 (0‐1.0)
OAB	6.0 (3.5‐7.0)	2.0 (0‐5.0)	7.50 (1.0‐10.75)	3.0 (1.5‐9.0)	5.0 (0.75‐7.75)	4.0 (1.0‐8.25)
LS	2.0 (0‐4.75)	0 (0‐2.0)	0 (0‐1.75)	0 (0‐0.5)	1.0 (0‐2.25)	0 (0‐2.0)

IQR, interquartile range; LS, low‐stream; LUT, Lower urinary tract; OAB, overactive bladder; SD, standard deviation; SUI, stress urinary incontinence; USP, Urinary symptom profile. Duration of bladder complaints is depicted in years.

* One patient had an indwelling catheter.

LUT symptoms were reported in 44 (86.3%) patients using the USP questionnaire. Higher median USP total scores and overactive bladder scores were found in the cohort of SCA1, SCA3, and SCA7 patients (Table 2). OAB symptoms were most prevalent, the commonest symptoms being urinary frequency and urgency (n = 34, 68%), and 26 patients (52.0%) presented both symptoms (Table S2). Nocturia (n = 15, 30%) and incontinence (n = 21, 42%) were also reported (Table S2).

**Figure 2:**
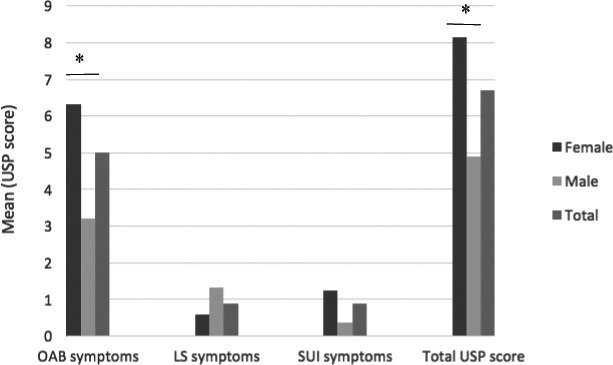
Lower urinary tract symptoms in 50 patients, depicted by gender, according to the Urinary symptom profile (USP) in terms of overactive bladder (OAB), stress urinary incontinence (SUI), low stream (LS) symptoms, and total score. *P < 0.05.

SUI was reported more in SCA3 patients (46.2% in SCA3 vs. 9.1% in all other SCAs, *P* = 0.028). A significantly higher mean USP score and OAB sub‐score were reported in females (8.14 vs. 4.91, *P* = 0.04, and 6.32 vs. 3.2, p < 0.01, respectively) (Figure 2). Low stream symptoms were more commonly reported by men (52.2% vs. 21.4%, *P* = 0.015).

To assess clinical determinants of LUT symptoms according to the USP score, a linear logistic regression was performed using age, gender, duration of ataxia, SARA score, and INAS count as covariates. None of these covariates were found to be a statistically significant determinant of LUT symptoms (*P* > 0.05).

### LUT symptom severity and impact on quality of life

LUT symptoms were most often classed as mild (n = 27, 61.4%) or moderate (n = 16, 32.7%) (Table S3) and only one patient (with SCA2) had severe LUT symptoms. There were no statistically significant differences between SCA subgroups. A total of 38 patients (77.6%) scored at least one point in the SF‐Qualiveen score, and SCA3 and SCA1 patients had higher impairment in QoL related to LUT symptoms. Spearman’s rank‐order correlations were run to determine the relationship between LUT‐related quality of life (SF‐Qualiveen) and clinical symptoms – a correlation between worse QoL related to LUT symptoms and worsening neurological disability in terms of SARA scores (*r* = 0.296, *P* = 0.041) and INAS count (*r* = 0.314, *P* = 0.038) was observed.

### Urological assessment and Urodynamic studies

Patients in the cohort were found to have additional pathology, namely benign prostate enlargement (n = 4), prostate adenocarcinoma (n = 1), and pelvic organ prolapse (n = 1) (Table S1). Twenty patients proceeded to urological assessment – 14 patients underwent uroflowmetry (SCA 7 (n = 4), SCA3 (n = 10)) and nine patients underwent cystometrogram (SCA 7 (n = 2), SCA3 (n = 7)) (Tables 3 and 4).

**Table 3:** Urodynamics findings in 10 patients with SCA3.

	Uroflowmetry				Cystometrography										
	Voided Volume(mL)	Pattern	Qmax (mL/s)	PVR (mL)	FDV (mL)	NDV (mL)	DO	Compliance	Total bladder capacity (mL)	Voided Volume (mL)	Pattern	Qmax (mL/s)	Pdet at Qmax (cm H_2_O)	PVR (mL)	BCI
1	74	intermittent	8.6	0	151	432	+	N	519	519	intermittent	14	30	0	100
2	NA	NA	NA	NA	225	NA	‐	reduced	225	0	‐‐‐	0	0	225	0
3	66	smooth	9	70	115	77	+	N	265	98	intermittent	2	33	167	43
4	550	smooth	24.6	0	281	308	+	N	460	410	intermittent	15	48	50	123
5	96	smooth	19	0	NA	NA	+	reduced	265	118	intermittent	8	49	0	89
6*	160	smooth	21.5	NA	141	141	+	N	180	NA	intermittent	NA	NA	NA	NA
7	NA	NA	NA	NA	135	195	+	N	650	625	intermittent	17.8	25	NA	149
8	179	smooth	19.4	175	NA	NA	NA	NA	NA	NA	NA	NA	NA	NA	NA
9	177	intermitent	23.2	325	NA	NA	NA	NA	NA	NA	NA	NA	NA	NA	NA
10	472	smooth	35.9	62	NA	NA	NA	NA	NA	NA	NA	NA	NA	NA	NA

BCI, bladder contractility index; DO, detrusor overactivity; FDV, first desire to void; N, normal; NA, not available; NDV, normal desire to void; Pdet at Qmax, detrusor pressure at maximum flow; PVR, postvoid residual volume; Q max, maximum flow; +, present; ‐, absent.

* Voiding study not possible in view of severe detrusor overactivity.

**Table 4:** Urodynamic findings in four patients with SCA7.

	Uroflowmetry				Cystometrography										
	Voided Volume (mL)	Pattern	Qmax (mL/s)	PVR(mL)	FDV (mL)	NDV (mL)	DO	Compliance	Total bladder capacity (mL)	Voided Volume (mL)	Pattern	Qmax (mL/s)	Pdet at Qmax (cmH_2_O)	PVR (mL)	BCI
1	224	intermittent	14	0	246	NA	‐	N	310	NA	intermittent	NA	34	20	NA
2	NA	NA	NA	23	105	125	‐	N	134	200	intermittent	7.2	59	47	95
3	99	intermittent	7.5	26	NA	NA	NA	NA	NA	NA	NA	NA	NA	NA	NA
4	59	NA	9.1	186	NA	NA	NA	NA	NA	NA	NA	NA	NA	NA	NA

BCI, bladder contractility index; DO, detrusor overactivity; FDV, first desire to void; N, normal; NA, not available; NDV, normal desire to void; Pdet at Qmax, detrusor pressure at maximum flow; PVR, postvoid residual volume; Q max, maximum flow; ‐, absent.

In patients with SCA 3 (Table 3), the mean postvoid residual volume (PVR) was 117.1 mL (SD 111.9) and the mean Qmax 14.0 mL/s (SD 9.6). Common findings were detrusor overactivity (n = 6; 85.7% – mean peak pressure 36.3 cmH_2_0), mean volume at first desire to void (FDV) 174.7 mL (SD 64.3) and the mean bladder capacity 366.3 mL (SD 176.9). During the voiding phase, the mean detrusor pressure at maximum flow (Pdet at Qmax) was 30.8 cmH_2_O (SD 18.0). The mean bladder contractility index (BCI) calculated using Schafer’s formula, which takes into account detrusor pressure at maximum flow and maximum flow rates, was 78.2 (SD 47.4), suggesting impaired detrusor contractility (weak < 100; normal 100‐159; strong > 150). An acontractile detrusor was reported in one patient. Using the bladder outlet obstruction (BOO) index, patients were found to be equivocal for obstruction (n = 2) or unobstructed (n = 3).

Patients with SCA7 (Table 4) had a mean PVR of 58.8 mL (SD 85.6). No patients were found to have detrusor overactivity, mean volume at first desire to void (FDV) was 175.5 mL (SD 99.7) and mean bladder capacity 294 mL (SD 226.3). Mean detrusor pressure at maximum flow (Pdet at Qmax) was 46.5 cmH_2_O (SD 17.7). Mean BCI was 69.5 (SD 36.1), suggesting impaired detrusor contractility. One patient was found to have obstructed voiding using the BOO index.

### Bowel symptoms and impact on quality of life

Bowel complaints were self‐reported by only six (11.8%) patients. Sixteen patients (31.4%) scored at least one point on the NBD scale and symptoms were classed as “very minor” (Table S4). Most patients (86.8%) achieved a score of 7 or higher on the NBD quality of life assessment, suggesting that their perceived bowel‐related quality of life was good. There was no correlation observed between LUT and bowel symptoms (*P* = 0.308), and no clinical determinants for the occurrence of bowel symptoms were identified.

### Management of LUT symptoms

At the time of assessment, seven (13.7%) patients were receiving nonpharmacological treatments, 11 (21.6%) patients were on oral agents for their bladder symptoms ‐ antimuscarinic agent (n = 9), beta‐3 receptor agonist, mirabegron (n = 1), and alpha‐blocker, tamsulosin (n = 1),‐ and two were undergoing percutaneous tibial nerve stimulation (PTNS). Only one patient was using an indwelling urethral catheter. Twelve patients (38.7%) self‐reporting LUT symptoms were not on pharmacological treatments. In these patients, the severity of symptoms was mostly graded as mild. Twenty‐six of the 44 patients (59.0%) with a positive USP score and twenty‐one of the 38 patients (55.3%) with some impact in QoL related to LUT symptoms were not on pharmacological treatment at the time of the survey.

## Discussion

In this cross‐sectional assessment of 51 patients with genetically confirmed SCAs, the prevalence of LUT symptoms was found to be high, however, only a small number were receiving treatment, suggesting that LUT symptoms were overlooked and undertreated.

Earlier studies using nonvalidated questionnaires have reported a lower prevalence of urinary dysfunction in SCA^7^, ^8^, however, this is not surprising as assessments were based on binary responses to generic questions in the INAS and clinical assessments (Table S5) ‐ the higher prevalence of LUT symptoms in our cohort could be due to the use of a standardized LUT questionnaire.

Overactive bladder symptoms, particularly urinary frequency and urgency, were the commonest ones in our series (68%) whereas incontinence was reported less frequently (42%). Our findings concur with previous reports in several non‐European cohorts in terms of OAB symptom predominance (Table S5). In SCA3, the most frequent LUT symptoms were nocturia (53.3 to 64%), urine incontinence (13.3 to 46%),^11^, ^12^ and urinary retention (in 54%).^11^ In a small series of nine patients with SCA6, increased urinary frequency and incontinence were reported in 33% of patients.^14^ In a Korean cohort of SCA patients, the prevalence of urinary incontinence in SCA2 and SCA6 was 12% and 20%, respectively.^8^ The complexity and severity of impairment of cerebellar dysfunction and other neurological systems are likely to influence the pattern and extent of LUT dysfunction, and this impairment is difficult to compare between different cohorts. Moreover, differences in the prevalence of LUT symptoms might be related to genetic and phenotypical differences between populations.^12^, ^25^

In our series, patients who self‐reported urinary complaints were older and had a more severe degree of ataxia. In the largest European cohort, older age was found to be a determinant for LUT symptoms in SCA2, whereas longer disease duration was a determinant for urinary dysfunction in SCA3 and SCA6.^7^

Our previous report of LUT symptoms in Friedreich’s ataxia likewise suggested the commonest OAB symptoms being urgency and frequency^26^ with comparable prevalence (80%). Urinary symptoms are highly prevalent in other neurodegenerative disorders, like synucleinopathies such as Multiple system atrophy (MSA)^27^ and Parkinson’s disease (PD) (ranging between 37 and 70%), however, the most commonly reported symptom is nocturia.^27^, ^28^, ^29^ Common to these disorders is a complex degeneration of the central systems controlling LUT, and the wide range of LUT symptoms may be put down to variable degrees of involvement of these systems.^30^

It is known that the cerebellum and basal ganglia play an important role in the regulation of the cerebral–brainstem neural circuit that facilitates the coordinated voluntary control of the voiding reflex, and these regions are extensively involved in SCAs.^25^ In addition, LUT dysfunction may occur due to multisite involvement. Findings of anal sphincter electromyography in patients with SCA3 and SCA6 demonstrate evidence for neurogenic changes, suggesting involvement of the Onuf’s nucleus of the sacral spinal cord.^14^, ^31^ Involvement of the Onuf’s nucleus and other anterior horn cells has also been shown in autopsy studies of SCA3.^32^ We were unable to depict a relation between LUT symptoms and the presence of extra‐cerebellar involvement (pyramidal, extrapyramidal, and peripheral nervous system involvement). This could be due to some heterogeneity of our SCA cohort, as it is composed of patients with a more pure cerebellar ataxia (SCA6) and others with a more widespread involvement. It appears that some SCA subtypes are more prone to urinary problems as reflected by higher median USP scores in SCA1, 3, and 7 patients. This trend and the contribution of different sites toward LUT dysfunction should be confirmed in larger samples of SCA patients in a setting of a multicenter study.

Our study demonstrates important differences in LUT symptoms between genders. The significance of this finding is uncertain in the absence of a control group, however, similar findings have been observed in women with mitochondrial disorders compared to healthy controls.^33^ Considering the older age of this cohort, patients’ previous medical history was registered to address additional urological and gynecological causes contributing to LUT symptoms, but the percentage of women with these diagnosis was residual, as the proportion of men with prostate pathology. A more in‐depth urological and gynecological observation should be considered in selected patients.

In our cohort, 13 patients (25%) were found to have LUT symptoms using questionnaires, however, this was not self‐reported. Though graded as mild in most cases, LUT symptoms were impacting quality of life, particularly in patients with greater neurological disability. 77.6% of patients reported an impact in urinary QoL, and these were found to be correlated with the severity of LUT symptoms (as assessed using I‐PSS) and degree of neurological disability (SARA scores and INAS), and therefore not only the LUT symptoms but also the functional impairment resulting from the neurological disease was contributing to their LUT symptom‐related QoL. Also, the pattern of LUT dysfunction (for instance, detrusor underactivity) might differently impair QoL ‐these differences should be explored in further studies. The impact on LUT‐related quality of life was comparable to other neurodegenerative disorders such as Friedreich ataxia and mitochondrial disorders.^26^, ^34^

One of the strengths of our study is the characterization of LUT dysfunction using urodynamic studies in SCA3 and SCA7 patients, allowing a more in‐depth understanding of LUT symptoms. The commonest finding was urinary storage dysfunction, due to detrusor overactivity. This is in line with two previous studies in SCA3 where detrusor overactivity was demonstrated only in 45%^13^ and 47% of patients.^31^ Overactivity, therefore, underpins the high prevalence of urinary storage symptoms reported using the USP questionnaire (Table 2). Interestingly though, detrusor overactivity was not seen in any of the SCA7 patients and different mechanisms might underpin storage symptoms in patients with SCA7. To our knowledge, this is the first study that reports urodynamics findings in SCA7 and a future study should use ambulatory urodynamics studies that permit a longer period of urodynamic testing over 3 hours. Importantly, detrusor pressures were not dangerously elevated to put the upper urinary tract at risk^33^ when compared with other neurological disorders such as *spina bifida* and spinal cord injury. This is only speculatory, as renal injury is uncommon in SCAs, and therefore longitudinal follow‐up of SCAs cohorts should include clinical and urodynamic assessments to explore the evolution of LUT dysfunction. The prevalence of voiding dysfunction was found to be high in terms of symptoms according to the USP questionnaire, and the observation of an intermittent urinary flow and elevated postvoid residual in most patients. Voiding dysfunction is therefore common in SCAs, however, postvoid residues were not elevated to the extent seen in MSA.^27^ Surprisingly, voiding dysfunction was most often due to an underactive detrusor rather than detrusor sphincter dyssynergia. The reasons for this remain unclear, altought it suggests that spinal cord pathology alone would not account for the voiding dysfunction. Previous studies have also demonstrated impaired or absent detrusor contractility in 27%^31^ and 29%.^13^ The cause for detrusor underactivity remains unexplicit, however, is reported in other neurological disorders such as Multiple sclerosis^35^ and PD,^36^ where it is related to worsening of motor function and quality of life. Future studies are needed to confirm this important observation and to explore neurological mechanisms for an underactive detrusor. Obstructed voiding was conclusively demonstrated in only one patient (SCA7) and therefore bladder outflow obstruction due to prostate enlargement was unlikely to be a cause for LUT symptoms in most men. Only videourodynamics or kinesiological sphincter EMG studies during urodynamics could confirm whether the obstructed voiding picture was due to detrusor sphincter dyssynergia. Additionally, SCA3 and SCA7 patients reported stress urinary incontinence in the USP questionnaire. The cause for stress incontinence is manifold and videourodynamics testing and sphincter EMG are useful tests to further evaluate whether denervation of the sphincter may be responsible.^37^

Bowel symptoms were uncommon, with little impact on quality of life. Bowel symptoms are not usually addressed in larger cohorts of SCA patients. Directed studies in SCA3 seem to also suggest a low prevalence of bowel complaints^11^, ^12^ and the largest study found a prevalence of constipation to be 13.3% and diarrhea 6.7%.^11^ Bowel complaints are reported with a greater prevalence in Freidreich’s ataxia, however, as high as 64%.^30^ Spinal cord involvement is less in SCAs compared to Friedreich’s ataxia, and this could account for the lower prevalence of bowel complaints in our cohort. Other neurodegenerative diseases with myelopathy, such as X‐linked adrenoleukodystrophy^38^, present higher prevalence and severity of bowel complaints. The presence of a more widespread central nervous system involvement in these other complex disorders in comparison with SCA patients might account for this difference. Also, it was previously reported in healthy subjects that exercise reduces colonic phasic activity, but later increases propagating activity, which may contribute to lesser resistance to colonic flow.^39^ Although physical activity was not systematically addressed, most patients in our SCA cohort were engaging in aerobic exercise and physiotherapy even with moderate disability, which could possibly contribute to the paucity of bowel symptoms.

This study highlights a significant treatment gap and an important percentage of symptomatic patients, both self‐reported (38.7%) and addressed by USP (59.0%), were not receiving treatment. There is also an expressive percentage of patients with an impact in QoL related to bladder symptoms not receiving treatment (55.3%). The varying degree of pelvic organ complaints and multitude of neurological symptoms is likely to leave LUT symptoms overlooked and undertreated. Further studies are needed to explore factors that contribute to this wide treatment gap, and explore how the LUT assessment can be embedded as an integral component of the evaluation of SCA patients. We emphasize the importance of a systematic enquiry of LUT symptoms and a multidisciplinary approach to management, as symptoms are amenable to treatment.^33^ As LUT symptoms are likely to progress during the course of the disease, it is imperative to continue enquiring about LUT symptoms at follow‐up visits.

The absence of a control group was a limitation to this study, however, the prevalence of LUT symptoms and the dysfunction were comparable to other study groups where a control population has been included.^34^ The survey was not designed to evaluate the relative contribution of neurological and urological pathology to LUT symptoms/ dysfunction and future studies should include specialist tests such as videourodynamics and sphincter EMG. Nevertheless, this study demonstrates that in a large cohort of patients with genetically confirmed SCAs, LUT storage and voiding symptoms are highly prevalent across different SCAs, in contrast to the mild degree of bowel symptoms. LUT symptoms are underrecognized by both patients and physicians, however, should be enquired about, as they impair quality of life and are amenable to treatment.

## Conflict of Interest

The authors decline any conflict of interest.

## Author Contributions

JAR collected data, performed statistical analysis, critical review of the results, draft generation. SS collected data, critical review of the results, draft generation. LDM collected data. TU collected data, critical review of the results. YTL collected data. NS collected data. HGM collected data, critical review of the draft. PG formulated the study, supervised data collection and analysis, critical review of the draft. JNP formulated the study, supervised data collection and analysis, generated the draft, and performed a critical review of the results.

## Supporting information


**Table S1.** Nonataxic signs and symptoms of SCA patients and relevant medical history, according to the presence of symptoms. Multiparity is defined as at least two vaginal deliveries. Prostate disease includes benign prostate enlargement and adenocarcinoma (1 pt). Numbers represent n (%). (total = 51 patients).
**Table S2.** Pattern of bladder symptoms in SCA patients – patients with scores ≥ 1 on the overactive bladder (OAB), low stream (LS) and stress urinary incontinence (SUI) score of the urinary symptom profile (USP) questionnaire. Numbers represent n (%). (total = 50 patients, 1 pt with indwelling catheter).
**Table S3.** International Prostate Symptom Score (I‐PSS) and SF‐Qualiveen (SF‐Q) questionnaires depiction according to SCA subtype. Severity of bladder symptoms in I‐PSS score was defined in mild [1‐7] and moderate [8‐19]. The last lines depict Spearmen correlation rank between SF‐Qualiveen and other LUT symptom scores – Urinary Symptom profile (USP) questionnaire and I‐PSS. (total = 49 patients).
**Table S4.** Description of self‐reported bowel complaints, and mean Neurogenic Bowel Dysfunction (NBD) score, classified as very minor – scores from 1 to 6. (total = 49 patients).
**Table S5.** Summary of most representative studies evaluating LUT symptoms in SCA patients.
