# Supplementary material for: Lower urinary tract and bowel dysfunction in spinocerebellar ataxias
Source: Ann Clin Transl Neurol. 2020 Dec 18;8(2):321–31. doi: 10.1002/acn3.51266 (PMC7886036; doi:10.1002/acn3.51266)
Supplement: Supplementary file 1 — Table S1. Nonataxic signs and symptoms of SCA patients and relevant medical history, according to the presence of symptoms. Multiparity is defined as at least two vaginal deliveries. Prostate disease includes benign prostate enlargement and adenocarcinoma (1 pt). Numbers represent n (%). (total = 51 patients). Table S2. Pattern of bladder symptoms in SCA patients – patients with scores ≥ 1 on the overactive bladder (OAB), low stream (LS) and stress urinary incontinence (SUI) score of the urinary symptom profile (USP) questionnaire. Numbers represent n (%). (total = 50 patients, 1 pt with indwelling catheter). Table S3. International Prostate Symptom Score (I‐PSS) and SF‐Qualiveen (SF‐Q) questionnaires depiction according to SCA subtype. Severity of bladder symptoms in I‐PSS score was defined in mild [1‐7] and moderate [8‐19]. The last lines depict Spearmen correlation rank between SF‐Qualiveen and other LUT symptom scores – Urinary Symptom profile (USP) questionnaire and I‐PSS. (total = 49 patients). Table S4. Description of self‐reported bowel complaints, and mean Neurogenic Bowel Dysfunction (NBD) score, classified as very minor – scores from 1 to 6. (total = 49 patients). Table S5. Summary of most representative studies evaluating LUT symptoms in SCA patients. [file ACN3-8-321-s001.pdf]

SUPPLEMENTARY MATERIAL

Supplementary Table 1 - Non-ataxic signs and symptoms of SCA patients and relevant medical history, according to presence of symptoms. Multiparity is defined as at least 2 vaginal deliveries. Prostate disease includes benign prostate enlargement and adenocarcinoma (1 pt). Numbers represent n (%). (total = 51 patients).

	**SCA1**	**SCA2**	**SCA3**	**SCA6**	**SCA7**	**TOTAL**
**Pyramidal features**						
Hyperreflexia	3 (75.0)	1 (10.0)	4 (30.8)	3 (17.6)	2 (33.3)	13 (26.0)
Babinsky sign	1 (25.0)	1 (10.0)	1 (7.7)	2 (11.8)	0	5 (10.0)
Spasticity	2 (50.0)	0	7 (53.8)	2 (11.8)	3 (50.0)	14 (28.0)
**Extrapyramidal features**	2 (50.0)	4 (40.0)	6 (46.2)	1 (5.9)	2 (33.3)	13 (26.0)
**Peripheral nervous system involvement**						
Distal hypo/arreflexia	1 (25.0)	8 (80.0)	8 (61.5)	8 (47.1)	1 (16.7)	26 (52.0)
Impaired vibration sense	3 (75.0)	7 (70.0)	11 (84.6)	10 (58.8)	1 (16.7)	32 (64.0)
Muscle atrophy	0	0	3 (23.1)	0	0	3 (6.0)
Fasciculations	0	1 (10.0)	2 (15.4)	0	0	3 (6.0)
**Relevant previous medical history**						
Multiparity	0	2 (18.2)	3 (23.1)	8 (47.1)	3 (50.0)	16 (31.4)
Pelvic organ prolapse	0	0	0	1 (5.9)	0	1 (2.0)
Prostate disease	1 (25.0)	1 (9.1)	0	2 (11.8)	1 (16.7)	5 (9.8)
**Vascular risk factors**						
Diabetes mellitus	0	1 (9.1)	0	4 (23.5)	1 (16.7)	6 (11.8)
Other risk factors (essential hypertension, dyslipidemia)	1 (25.0)	1 (9.1)	2 (15.4)	6 (35.3)	1 (16.7)	11 (21.6)

Supplementary Table 2 – Pattern of bladder symptoms in SCA patients - patients with scores ≥ 1 on the overactive bladder (OAB), lowstream (LS) and stress urinary incontinence (SUI) score of the urinary symptom profile (USP) questionnaire. Numbers represent n (%). (total = 50 patients, 1 pt with indwelling catheter).

	**SCA1**	**SCA2**	**SCA3**	**SCA6**	**SCA7**	**TOTAL**
**OAB score (≥ 1 point)**	4 (100)	7 (63.6)	10 (83.3)	15 (88.2)	5 (83.3)	41 (82.0)
Frequency	4 (100)	6 (54.6)	8 (66.7)	13 (76.5)	3 (50.0)	34 (68.0)
Urgency	4 (100)	6 (54.6)	9 (75.0)	10 (58.8)	5 (83.3)	34 (68.0)
Incontinence	1 (25.0)	3 (27.3)	7 (58.3)	7 (41.2)	3 (50.0)	21 (42.0)
Polyuria	2 (50.0)	2 (18.2)	5 (41.7)	5 (29.4)	0	14 (28.0)
Nocturia	2 (50.0)	1 (9.1)	4 (33.3)	6 (35.3)	2 (33.3)	15 (30.0)
Nocturnal incontinence	0	3 (27.3)	1 (8.3)	2 (11.8)	0	6 (12.0)
**LS score (≥ 1 point)**	2 (50.0)	4 (36.4)	4 (33.3)	4 (23.5)	4 (66.7)	18 (36.0)
Voiding	1 (25.0)	0	3 (25.0)	0	2 (33.3)	6 (12.2)
Urinary Flow	2 (50.0)	2 (18.2)	2 (16.7)	1 (5.9)	2 (33.3)	9 (18.4)
Stream	2 (50.0)	4 (36.4)	2 (16.7)	4 (23.5)	1 (16.7)	13 (26.5)
**SUI (≥ 1 point)**	1 (25.0)	1 (9.1)	6 (50.0)	5 (29.4)	3 (50.0)	16 (32.0)

Supplementary Table 3 – International Prostate Symptom Score (I-PSS) and SF-Qualiveen (SF-Q) questionnaires depiction according to SCA subtype. Severity of bladder symptoms in I-PSS score was defined in mild [1-7] and moderate [8-19]. The last lines depict Spearmen correlation rank between SF-Qualiveen and other LUT symptom scores – Urinary Symptom profile (USP) questionnaire and I-PSS. (total =49 patients).

	**SCA1**	**SCA2**	**SCA3**	**SCA6**	**SCA7**	**TOTAL**
**I-PSS** mean (SD)	8.5 (3.79)	4.91 (6.20)	6.8 (4.02)	4.94 (3.13)	6.20 (5.17)	5.8 (4.41)
**Mild [n(%)]**	2 (50.0)	6 (54.5)	6 (50.0)	11 (64.7)	2 (33.3)	27 (55.1)
**Moderate [n(%)]**	2 (50.0)	2 (18.2)	5 (41.7)	5 (29.4)	2 (33.3)	16 (32.7)
**SF-Q overall** mean (SD)	1.44 (0.55)	0.59 (0.95)	1.43 (1.2)	0.70 (0.80)	1.10 (1.29)	0.96 (1.02)
Bothers	0.75 (0.29)	0.5 (0.95)	1.25 (1.27)	0.38 (0.67)	0.92 (1.07)	0.70 (0.97)
Fears	2.0 (0.70)	0.727 (1.08)	1.7 (1.60)	0.72 (0.91)	1.25 (1.25)	1.10 (1.23)
Feelings	1.13 (0.63)	0.545 (0.907)	1.58 (1.43)	0.47 (0.81)	1.08 (1.20)	0.87 (1.10)
Frequency	1.89 (1.25)	0.591 (1.02)	1.13 (0.98)	1.25 (1.28)	1.17 (1.83)	1.09 (1.20)
**SF-Qualiveen (≥ 1 point) n (%)**	4 (100)	5 (45.5)	1 (91.7)	14 (87.5)	4 (66.7)	38 (77.6)
**Spearman rank order correlation**						
		I-PSS		USP score		
**SF-Qualiveen**		Positive correlation *r*=0.583; p<0.001		Positive correlation *r*=0.806; p<0.001		

SD: standard deviation.

Supplementary table 4 – Description of self reported bowel complaints, and mean Neurogenic Bowel Dysfunction (NBD) score, classified as very minor – scores from 1 to 6. (total = 49 patients).

	**SCA1**	**SCA2**	**SCA3**	**SCA6**	**SCA7**	**TOTAL**
**Self-reported bowel complaints n (%)**	1 (25.0)	1 (10.0)	2 (15.4)	1 (5.9)	1 (16.7)	6 (12.0)
**NBD** Mean (SD)	0.75 (1.5)	0.60 (1.27)	1.17 (2.21)	0.65 (1.46)	1.0 (1.55)	0.82 (1.6)
**Very minor [n(%)]**	4 (100)	10 (100)	12 (100)	17 (100)	6 (100)	49 (100)
**Pt scoring more than 1 point in NBD**	1 (25.0)	3 (30.0)	3 (25.0)	6 (35.3)	3 (50.0)	16 (32.7)
**QoL related to bowel symptoms – NBD score.** Mean (SD)	7.7 (1.5)	9.9 (0.35)	9.0 (1.67)	8.7 (1.6)	9.4 (0.89)	9.03 (1.42)

SD: standard deviation; Pt: patients; QoL: quality of life.

Supplementary table 5 – Summary of most representative studies evaluating LUT symptoms in SCA patients.

**Study**	**Type of SCA**					**Comments**
	SCA1	SCA2	SCA3	SCA6	SCA7	
**Schmitz-Hubsch T, *et al.*[7]**	35%	40.4%	45.6%	31.1%		Binary question INAS
**Lee WY, *et al.*[8]**	17%	12%	13%	20%	0%	% of urinary incontinence by clinical assessment
**Jang M, *et al.*[9]**	9.0%	7.8%	7.7%	15.0%	50.0%	ICS questionnaires.
**Schols L, *et al.*[10]**			Urge incontinence 19%			Clinical assessment
**França Jr MC, *et al.*[11]**			Nocturia (64%)<br>Urine retention (54%).			Comprehensive questionnaire
**Yeh TH, *et al.*[12]**			Nocturia (53.3%); urine incontinence (13.3%)			Comprehensive questionnaire
**Musegante AF, *et al.*[13]**			17 (13.9%) - urgency (12.3%), incontinence (7.3%)			Comprehensive questionnire
**Tateno F, *et al.*[14]**				56%<br>Urinary frequency and incontinence (33%);<br>Urinary urgency (22%)		Comprehensive questionnaire
**Pedroso J.L., *et al.*[15]**		27.27%				Binary question INAS
